# Hyperphosphatemia Is an Independent Risk Factor for Mortality in Critically Ill Patients: Results from a Cross-Sectional Study

**DOI:** 10.1371/journal.pone.0133426

**Published:** 2015-08-07

**Authors:** Dominik G. Haider, Gregor Lindner, Michael Wolzt, Sufian S. Ahmad, Thomas Sauter, Alexander Benedikt Leichtle, Georg-Martin Fiedler, Valentin Fuhrmann, Aristomenis K. Exadaktylos

**Affiliations:** 1 Department of Emergency Medicine, Inselspital, University Hospital Bern, Freiburgstrasse, Bern, Switzerland; 2 Department of Clinical Pharmacology, Medical University of Vienna, Spitalgasse 23, Vienna, Austria; 3 Center of Laboratory Medicine, Inselspital, University Hospital Bern, Bern, Switzerland; 4 Department of Intensive Care Medicine, University Hospital Hamburg-Eppendorf, Martinistrasse 52, Hamburg, Germany; University of Leicester, UNITED KINGDOM

## Abstract

**Background:**

Phosphate imbalances or disorders have a high risk of morbidity and mortality in patients with chronic kidney disease. It is unknown if this finding extends to mortality in patients presenting at an emergency room with or without normal kidney function.

**Methods and Patients:**

This cross sectional analysis included all emergency room patients between 2010 and 2011 at the Inselspital Bern, Switzerland. A multivariable cox regression model was applied to assess the association between phosphate levels and in-hospital mortality up to 28 days.

**Results:**

22,239 subjects were screened for the study. Plasma phosphate concentrations were measured in 2,390 patients on hospital admission and were included in the analysis. 3.5% of the 480 patients with hypophosphatemia and 10.7% of the 215 patients with hyperphosphatemia died. In univariate analysis, phosphate levels were associated with mortality, age, diuretic therapy and kidney function (all p<0.001). In a multivariate Cox regression model, hyperphosphatemia (OR 3.29, p<0.001) was a strong independent risk factor for mortality. Hypophosphatemia was not associated with mortality (p>0.05).

**Conclusion:**

Hyperphosphatemia is associated with 28-day in-hospital mortality in an unselected cohort of patients presenting in an emergency room.

## Introduction

Electrolyte disorders frequently develop in critically ill patients. In patients with chronic kidney disease (CKD), there is derangement of phosphate homeostasis. As CKD progresses, phosphate excretion by the kidney diminishes, which raises the serum phosphate level. This positive phosphate balance is further exacerbated by the impairment of the reservoir function of the skeleton in advanced CKD [1]. Observational studies in end-stage kidney disease show a graded relationship between serum phosphate level and cardiovascular events [2,3]. Similar observations have been made in patients with advanced CKD who are not on dialysis [4,5]. Even in people with normal kidney function, a relative increase in serum phosphate within the normal range has been linked to cardiovascular disease in a number of observational cohorts, prompting some to suggest that phosphate may be the “the new cholesterol” [6–8].

In the general hospital population, the prevalence of moderate hypophosphatemia ranges between 2.2 and 3.1% [9,10], and the prevalence of severe hypophosphatemia is reported to be 0.2 to 0.4% [11,12]. One study reports that 45% of all hospital hypophosphatemia cases occur in an ICU population [13]. Hypophosphatemia has a higher incidence in certain patient groups, such as patients with diabetic ketoacidosis, sepsis, and postoperative patients. Hypophosphatemia is found in as many as 34% of patients after elective cardiac surgery [14]. An extremely high incidence of hypophosphatemia is reported after major hepatic surgery, where almost all patients develop hypophosphatemia in the first postoperative week [15,16].

In this retrospective study, we have tested whether imbalances in systemic phosphate concentrations are associated with in-hospital mortality in unselected patients presenting at an ER.

## Materials, Methods and Patients

All patients admitted to the ER of the Inselspital, University Hospital Bern, between 1 January 2009 and 31 December 2010 were included in this cross-sectional analysis. During the study period, 22,239 patients were enrolled in the study. In the case of multiple admissions, only the first admission to the ER was considered for the analysis.

For these 22,239 patients, data on age, sex, admission type (medical or surgical), pre-existing diuretic medication, country of residence, hospital admission, length of hospital stay, outcome and final diagnosis—as classified by the *International Classification of Diseases*, 10th revision (ICD-10)—were collated.

Patients who had at least one cardiovascular, neurological or metabolic, nephrologic, intestinal or hepatic comorbidity alongside the primary reason for admission received a blood test including Phosphate concentration.

Plasma phosphate concentrations ≤0.75 mmol/l were considered as hypophosphatemia, concentrations between 0.75 and 1.45 as normal/physiological levels and ≥1.46 mmol/l as hyperphosphatemia.

A waiver for informed consent was provided for this retrospective analysis of pseudononymized data. Data were analyzed anonymously. Due to ethical and legal restrictions in accordance with the guidelines provided by the ethical committee of Bern data are available upon request from the authors who may be contacted at the corresponding author address, the Department of Emergency Medicine at the University Hospital of Bern. The study protocol was approved by the Ethics Committee of the Canton of Bern, Switzerland.

## Statistical Analysis

Data are presented as medians (IQR) or proportions, as appropriate. Between-group comparisons of continuous variables were performed using the Mann–Whitney U test.

Pearson’s Chi-Square Test was used for the identification of associations with survival.

Cox regression analysis was used to explore the association of the various predictors with the presence of electrolyte disorders and with hospitalization. Predefined covariates were added to the logistic regression models. Cox regression was used to test associations of the diuretics with the survival time adjusted for predefined covariates.

A two-sided *P* value of <0.05 was considered statistically significant for all analyses. The statistical analysis was performed using SPSS (SPSS for Windows V.17.0, Chicago, IL, USA).

## Results

**Fig 1:**
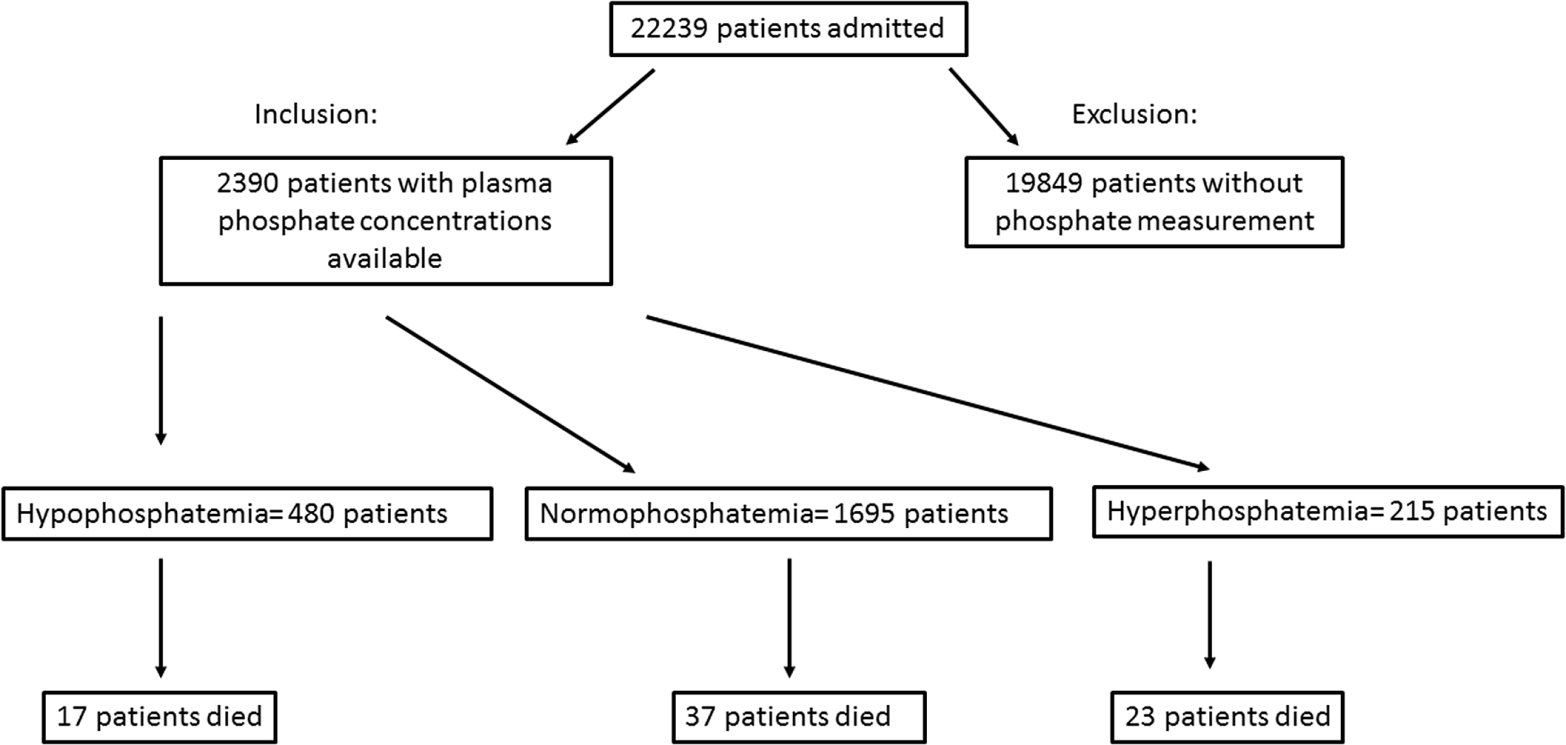
Patient flow chart.

22,239 patients admitted to the ER during the observation period were screened for alterations in systemic phosphate levels (Fig 1). 19,849 patients were excluded because no phosphate concentrations were available from laboratory reports on hospital entry and 2,390 patients were included in the analysis (Fig 1). Patients had a median age of 57 (range 40 to 71) years and 1399 (58.5%) were male. Baseline characteristics are shown in Table 1 and Table 2. Median length of hospital stay was 3 (range 0 to 9) days and 28-day in-hospital mortality was 3.2% (n = 77).

**Table 1:** Baseline characteristics of patients on hospital admission (n = 2390). Medians with IQRs.

Parameter	Median (IQR)
Length of hospital stay (days)	3 (0;9)
Age (years)	57 (40;71)
eGFR (MDRD) (ml/min/1,73m2)	89.4 (57.4;114.4)
Sodium (mmol/l)	139 (136;141)
Chloride (mmol/l)	103 (99;106)
Phosphate (mmol/l)	1.9 (0.8;1.2)
Calcium (mmol/l)	2.25 (2.15;2.34)
Potassium (mmol/l)	3.9 (3.7;4.3)
Magnesium (mmol/l)	0.8 (0.72;0.88)
Osmolality (mosm/kg)	291 (283;306)

**Table 2:** Baseline characteristics of patients on hospital admission (n = 2390).

Parameter	Total number (n =)
Ethnicity (African/Caucasian)	27/2363
Mortality	77
Patients with diuretic therapy	510
Sex (m/f)	1399/991

When compared with patients with physiological phosphate levels, patients with hyperphosphatemia were 8 years older (57 vs. 65 years, p<0.05), stayed 3 more days in hospital, had lower eGFR, sodium, chloride and calcium but higher potassium, magnesium and osmolality (Table 3). On average, patients with hypophosphatemia were 3 years younger than patients with physiological phosphate levels (54 years, p<0.05) and their systemic magnesium, potassium and osmolality also differed (Table 3).

**Table 3:** Data is presented as mean values with IQRs.

	Hypophosphatemia	Normophosphatemia	Hyperphosphatemia
Length of hospital stay (days)	3 (0;8)	3 (0;3)	6 (1;14)*
eGFR(MDRD) (ml/min/1,73m2)	90.7 (65.5;114.3)	92.8 (64.0;92.7)	22.4 (11.7;64.6)*
Sodium (mmol/l)	139 (135;141)	139 (136;139)	137 (133;140)*
Chloride (mmol/l)	104 (100:106)	103 (100;103)	101 (96;106)*
Phosphate (mmol/l)	0.68 (0.57;0.75)*	1.04 (0.93;1.04)	1.83 (1.63;2.23)*
Calcium (mmol/l)	2.24 (2.13;2.34)	2.26 (2.17;2.26)	2.17 (2.05;2.23)*
Potassium (mmol/l)	3.8 (3.5;4.1)*	4.0 (3.7;4.0)	4.4 (3.9;5.2)*
Magnesium (mmol/l)	0.77 (0.68;0.83)*	0.81 (0.73;0.81)	0.93 (0.81;1.04)*
Osmolality (mosm/kg)	288 (282;293)*	291 (283;291)	311 (295;328)*

Statistical testing shows group comparison of patients with hypophosphatemia (n = 480) and hyperphosphatemia (n = 215) versus patients with serum phosphate within the normal range (n = 1695) (Mann-Whitney U, p<0.05*).

In univariate analysis, phosphate levels were strongly associated with mortality and eGFR (Table 4). In a multivariate Cox regression model, only hyperphosphatemia was an independent risk factor for mortality (Table 5). In contrast, hypophosphatemia was not associated with mortality in multivariate Cox regression (Table 6). In this group of patients, only eGFR was an independent risk factor for mortality (Table 6). For ethnicity no statistical result could be obtained as too few cases were included (Tables 5 and 6).

**Table 4:** Associations of phosphate levels with different parameters in univariate analysis.

Parameter	p-value
Sex	0.054
**Mortality**	<0.001*
Ethnicity	0.91
**Diuretic therapy**	<0.001*
**Age**	<0.01*
**eGFR**	<0.001*

Pearson’s Chi-Square Test (p<0.05*).

**Table 5:** Multivariate Cox regression analysis for mortality in patients with hyperphosphatemia (A) and patients with hypophosphatemia (B).

Parameter	OR (95% CI)	p-value
**Hyperphosphatemia**	**3.29 (1.8;6.1)**	<0.001*
Sex	0.29 (0.8;2.3)	0.34
Age	1.01 (0.9;1.0)	0.07
Diuretic therapy	0.7 (0.4;1.3)	0.22
eGFR	1.0 (0.9;1.0)	0.83
Ethnicity	n.a.	n.a.

(p<0.05*), data presented in odds ratios (OR) with 95% confidence intervals (95% CI).

**Table 6:** Multivariate Cox regression analysis for mortality in patients with with hypophosphatemia.

Parameter	OR (95% CI)	p-value
Hypophosphatemia	1.24 (0.66;2.30)	0.51
Sex	1.28 (0.75;1.28)	0.36
Age	1.01 (0.99;1.03)	0.10
Diuretic therapy	0.75 (0.42;1.35)	0.33
**eGFR**	**1.001 (1.00;1.002)**	0.008*
Ethnicity	n.a.	n.a.

(p<0.05*), data presented in odds ratios (OR) with 95% confidence intervals (95% CI).

**Fig 2:**
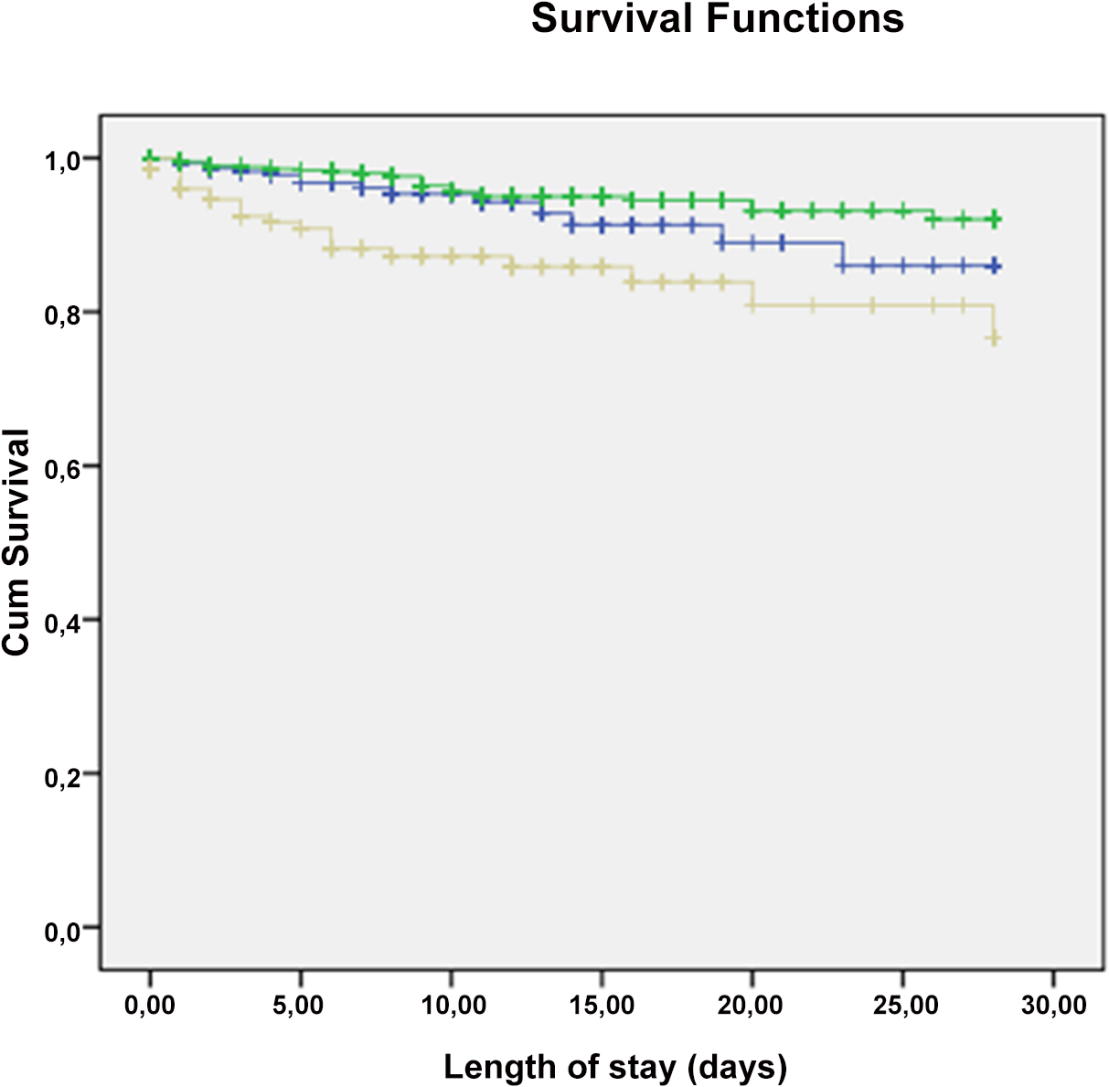
Kaplan-Meier curve for 28 day in-hospital mortality in patients with hyperphosphatemia (grey line) versus patients with hypophosphatemia (blue line) and patients with normal phosphate levels (green line) (p<0.001).

The Kaplan Meier curve shows that the increase in mortality in patients with hyperphosphatemia becomes apparent between days 2 and 5 after hospital admission (Fig 2).

## Discussion

Our study demonstrates that hyperphosphatemia is an independent predictor for mortality in patients presenting at an ER. In patients with hypophosphatemia, only eGFR remains independently associated with mortality.

Hyperphosphatemia has been repeatedly linked to CKD [1–8].While hyperphosphat-emia may be a tangible indicator of deteriorating kidney function, it appears that lack of phosphate homeostasis may also be associated with the increased risk of cardiovascular events and mortality that has become a hallmark of CKD [2–6]. The need to maintain phosphorus concentrations within a recommended range is reflected in evidence-based guidelines. However, no studies have been performed to investigate the influence of phosphate levels on the outcomes of patients presenting at an ER. Although we detected strong associations with kidney function, hyperphosphatemia remained an independent risk factor for mortality in the multivariate analyses. There is evidence that phosphate additives in food may harm the health of persons with normal renal function [17]. More recent studies have shown that the association between high phosphate concentrations and higher mortality is not restricted to persons with renal disease; it can also be observed in persons with cardiovascular disease and even in the general population. High-normal serum phosphate concentrations are associated with coronary calcification in young, healthy men and were found to be a predictor of cardiovascular events in the Framingham study [18,19]. Elevated mortality in association with high-normal serum phosphate concentrations was seen mainly among persons with cardiovascular disease who had normal renal function [20]. Our results strengthen these findings and emphasize that hyperphosphatemia is an electrolyte disorder that should be controlled in patients presenting at an ER.

In the present study, we found no association between hypophosphatemia and mortality. On the other hand, many other studies have found an association between hypophosphatemia and increased mortality [13,14,21–28]. Severe hypophosphatemia has been reported to predict an increase of up to 8-fold in mortality in sepsis patients [21]. However, hypophosphatemia has not been associated with increased mortality after cardiac surgery and in diabetic ketoacidosis [14,23]. Overall, it therefore remains unclear whether hypophosphatemia actually contributes to mortality, or is merely a marker for severe illness.

In summary, hyperphosphatemia is an independent predictor for mortality in an unselected patients cohort presenting at an ER. Hypophosphatemia was not associated with mortality in these patients.
